# Self-assembling bilayer wiring with highly conductive liquid metal and insulative ion gel layers

**DOI:** 10.1038/s41598-023-32580-x

**Published:** 2023-04-12

**Authors:** Koki Murakami, Yuji Isano, Juri Asada, Natsuka Usami, Yutaka Isoda, Tamami Takano, Ryosuke Matsuda, Kazuhide Ueno, Ohmi Fuchiwaki, Hiroki Ota

**Affiliations:** 1 Department of Mechanical Engineering, Yokohama National University, 79-5, Tokiwadai, Hodogaya-Ku, Yokohama, Kanagawa 240-8501 Japan; 2 Department of Chemistry and Life Science, Yokohama National University, 79-5, Tokiwadai, Hodogaya-Ku, Yokohama, Kanagawa 240-8501 Japan; 3 Graduate School of System Integration, Yokohama National University, 79-5, Tokiwadai, Hodogaya-Ku, Yokohama, Kanagawa 240-8501 Japan; 4 Graduate School of Engineering, Yokohama National University, 79-5, Tokiwadai, Hodogaya-Ku, Yokohama, Kanagawa 240-8501 Japan

**Keywords:** Electrical and electronic engineering, Mechanical engineering

## Abstract

Ga-based liquid metals (LMs) are expected to be suitable for wiring highly deformable devices because of their high electrical conductivity and stable resistance to extreme deformation. Injection and printed wiring, and wiring using LM–polymer composites are the most popular LM wiring approaches. However, additional processing is required to package the wiring after LM patterning, branch and interrupt wiring shape, and ensure adequate conductivity, which results in unnecessary wiring shape changes and increased complexity of the wiring methods. In this study, we propose an LM–polymer composite comprising LM particles and ion gel as a flexible matrix material with low viscosity and specific gravity before curing. Moreover, the casting method is used for wire patterning, and the material is cured at room temperature to ensure that the upper insulative layer of the ion gel self-assembles simultaneously with the formation of LM wiring in the lower layer. High conductivity and low resistance change rate of the formed wiring during deformation are achieved without an activation process. This ion gel–LM bilayer wiring can be used for three-dimensional wiring by stacking. Furthermore, circuits fabricated using ion gel–LM bilayer wiring exhibit stable operation. Therefore, the proposed method can significantly promote the development of flexible electronic devices.

## Introduction

With the advent of soft robotics^1–3^ and wearable devices^4–6^, demand for soft electronics using flexible materials such as Ecoflex, polydimethylsiloxane (PDMS), and gels as substrates has been steadily increasing. Generally, such devices require highly deformable conductive materials such as nanocomposite elastomers embedded with silver nanowires^7^, poly(3,4-ethylenedioxythiophene) poly(styrenesulfonate) (PEDOT:PSS)^8,9^, and carbon nanotubes (CNTs)^10^. Among them, liquid metals (LMs), which are liquids at room temperature, have attracted significant attention. For instance, the Ga-based alloy Galinstan (68 wt% Ga, 22 wt% In, and 10 wt% Sn) is a biocompatible material with a high electrical conductivity of $3.46\times {10}^{6}$ S/m^11^, melting point of 11 °C^12^, and viscosity of $2.4 \mathrm{mPa}\cdot\mathrm{s}$^13^. This liquid material has been used for soft and stretchable device wiring^14^, sensor reactors^15,16^, connections with different conductive materials^17^, bioelectrodes^18,19^, actuators^20,21^, and dielectric elastomers^22^. Although Galinstan is more difficult to process than other conductive materials due to its high surface tension (~ 535 mN/m)^23^, it has a much lower Young's modulus and smaller resistance change due to deformation. Therefore, LMs, such as Galinstan, are conductive materials with a high affinity for flexible materials that can withstand extreme deformations, such as biological tissues and gels.

Conventional technologies for wiring LMs, such as direct drawing^24–26^, screen printing^27,28^, activation by sintering^29–31^ and transfer^19,32^ have been reported. These technologies leave the LM bare on the substrate and require an encapsulation process to prevent its destruction and leakage after wiring. However, this encapsulation process complicates the wiring method; consequently, reconfiguring the device becomes difficult because repairing or correcting the position of the elements and wiring after encapsulation is difficult.

Injection into a premade channel^15,33,34^ and sedimentation of LM in silicone rubber^35,36^ have also been proposed to integrate the encapsulation and wiring processes. However, fabricating interrupted or branched shapes using injection wiring is difficult. In the second method, the wiring is electrically insulating because silicone rubber fills up the space between LM particles, requiring under pressure sintering to form a percolation network that ensures conductivity. This additional activation process may cause LM seepage and unwanted changes in the wiring shape.

Similarly, conventional LM–polymer composites are electrically insulating by forming polymer walls that separate the LM particles. LM particle accumulation and sedimentation are obstructed by the high viscosity and specific gravity of the polymer, which increases the inter-particle distance and necessitates an additional activation process to ensure conductivity^35,37^. Therefore, the physical properties of the polymer mixed with LM must be optimized to achieve high conductivity without activation.

In this study, we propose a method to realize LM wiring without additional activation processes. In addition, we use an ion gel containing an ionic liquid as a flexible matrix material to obtain an electronically insulative layer that self-assembles atop the LM wiring^38^. The solution is prepared by mixing LM, ionic liquid, and polymer in a solvent, and is patterned on a substrate by a casting method. The LM is sedimented before the ion gel cures, resulting in a self-assembled bilayer structure with a bottom conductive layer of LM–ion gel composite (LMGC) and a top insulative layer of the ion gel. Owing to the physical properties of the ion gel, the LM particles formed during mixing come into contact after sedimentation without any activation process, resulting in high electrical conductivity. Simultaneously, the self-assembly of the top ion gel forms an insulative layer over the wiring without an encapsulation process. This study serves as a foundation for developing easier fabrication methods for circuit-mounted devices with LM wiring.

## Results

### Ion gel–LM bilayer

**Figure 1:**
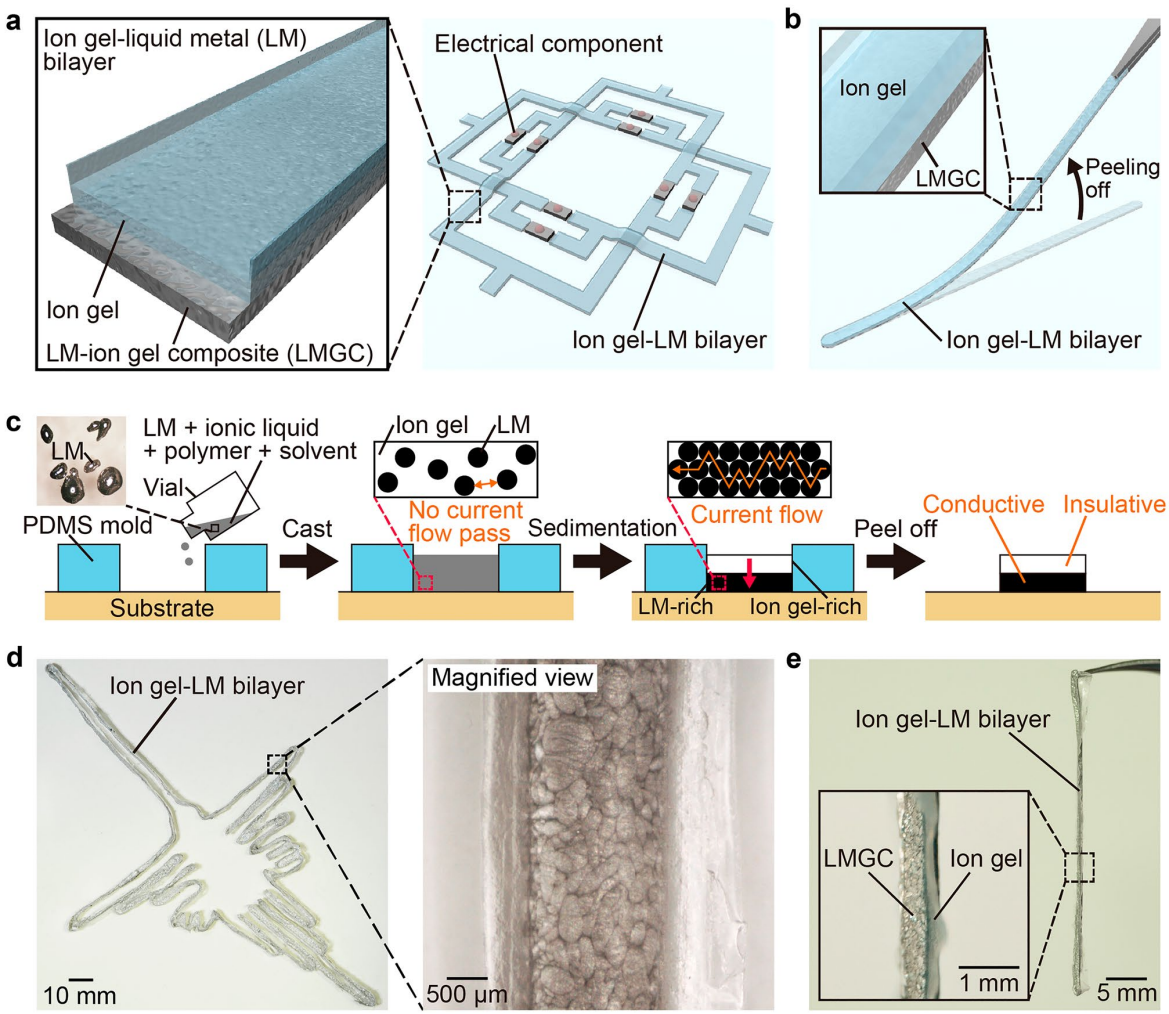
Patterning of the ion gel-liquid metal (LM) bilayer. (**a**) Schematic of the ion gel–LM bilayer. The bilayer structure, consisting of the bottom LM–ion gel composite (LMGC) conductive layer and the top ion gel insulating layer, is self-assembled. (**b**) Schematic of the ion gel–LM bilayer being peeled off with tweezers. (**c**) Fabrication method of the ion gel–LM bilayer. The LM in the solution sediments until the solvent volatilizes, resulting in the self-assembly of the top ion gel-rich layer and the bottom LM-rich layer after the gelation of the ion gel is completed. (**d**) The ion gel–LM bilayer patterned in the desired shape. (**e**) The ion gel–LM bilayer lifted up. The LM was retained without seepage.

Figure 1a shows a schematic of the ion gel–LM bilayer fabricated in this study. As shown in Fig. 1a, the conductive LMGC layer at the bottom and the insulative ion gel layer at the top self-assembled to complete the encapsulation and wiring simultaneously. As shown in Fig. 1b, the ion gel–LM bilayer can be lifted with tweezers after wiring and easily moved.

Figure 1c shows the fabrication method for the ion gel–LM bilayer in this study. Galinstan, a gallium alloy with high biocompatibility, was used as the LM material. First, the poly(vinylidene fluoride hexafluoropropylene) (PVDF-HFP) polymer, ionic liquid, and LM were mixed in methyl ethyl ketone (MEK) solvent and stirred at 1500 rpm for 60 s using a magnetic stirrer to form LM particles with an average diameter of 167 μm in the solution. The solution was cast into a PDMS mold attached to a substrate for patterning. The LM in the solution sedimented until the solvent volatilized and ion gel gelation was completed, resulting in the self-assembly of the ion gel-rich layer at the top and the LM-rich layer at the bottom. Subsequently, the ion gel–LM bilayer was fabricated by removing the composite gel from the PDMS mold. Figure 1d shows the actual ion gel–LM bilayer. As shown in Fig. 1d, an ion gel–LM bilayer with the desired pattern was fabricated. The ion gel–LM bilayer can be lifted with tweezers, as shown in Fig. 1e, and the LM is retained without dripping.

### Fundamental characteristics of ion gel–LM bilayer

**Figure 2:**
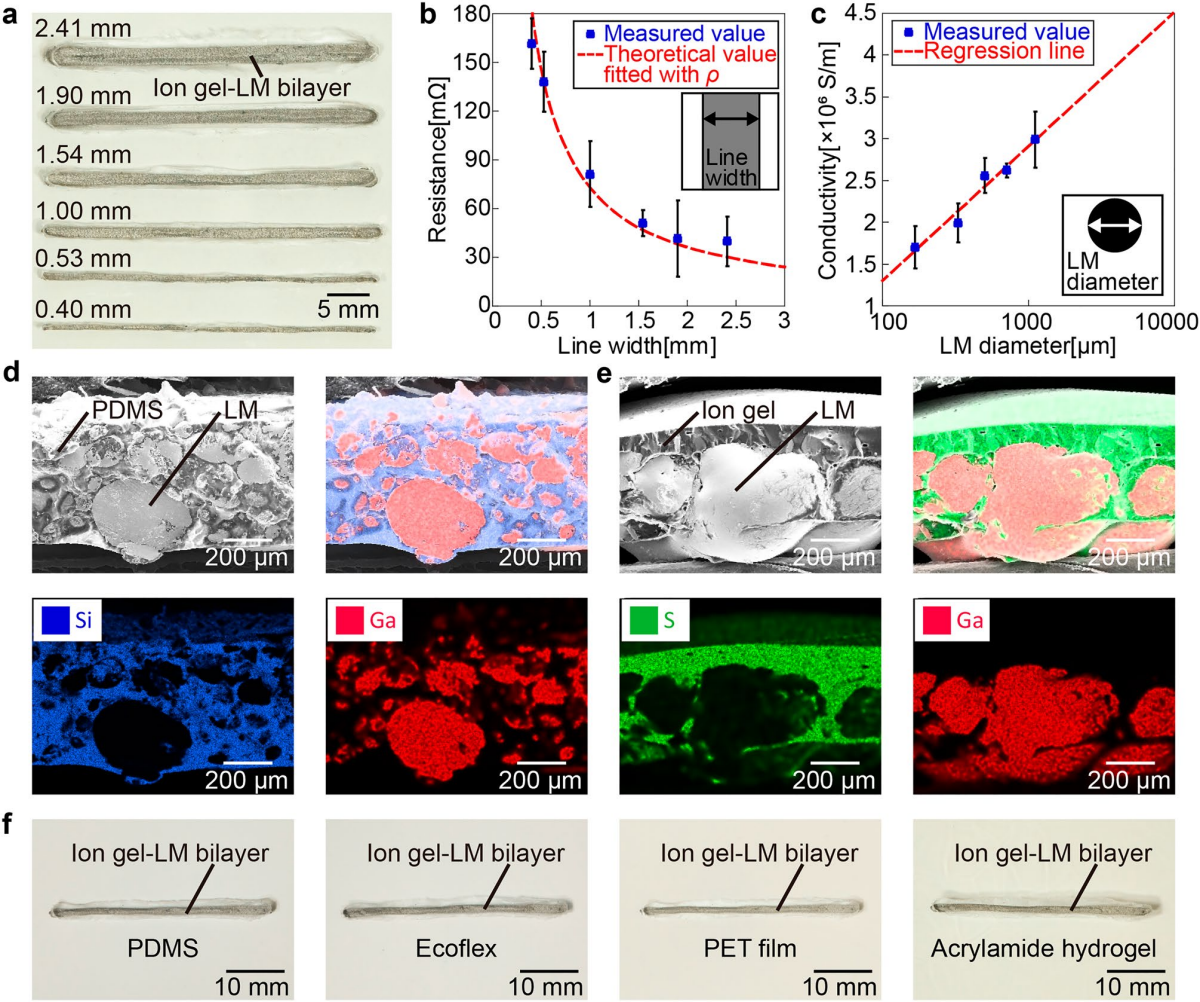
Fundamental characteristics of the ion gel–LM bilayer. (**a**) The ion gel–LM bilayer wirings with various linewidths. The minimum linewidth was 404 μm. (**b**) Relationship between linewidth and resistance value of the ion gel–LM bilayer wiring. The resistance value decreases as a function of the linewidth. (**c**) Relationship between LM particle diameter and resistance value of the ion gel–LM bilayer wiring. The resistance value decreases as a function of the LM particle diameter. (**d**) Cross-sectional images of PDMS–LM composite using scanning electron microscopy-energy dispersive X-ray spectroscopy (SEM EDX). (**e**) Cross-sectional images of the ion gel–LM bilayer using SEM EDX. (**f**) The ion gel–LM bilayer wirings on PDMS, Ecoflex, PET film, and acrylamide hydrogel. The ion gel–LM bilayer wiring can be applied to various flexible substrates.

Figure 2a shows the ion gel–LM bilayer wiring on a glass substrate with the same thickness and various linewidths. The smallest linewidth that could be fabricated is 404 μm. A magnified view of each ion gel–LM bilayer wiring is shown in Supplementary Fig. S1. The relationship between the linewidth designed by the PDMS mold and the actual measured linewidth of the ion gel–LM bilayer wiring is shown in Supplementary Fig. S2. The measured values of the linewidth are, on average, 494 μm smaller than the designed values because of the removal of the MEK solvent during gelation. The correlation coefficient between the designed and measured linewidths is 0.998. Figure 2b shows the relationship between the linewidth and resistance for a 42 mm length of the ion gel–LM bilayer wiring. The theoretical value of the resistance is given as following (Eq. (1)).

1 $$R=\rho \frac{L}{S}$$

where $R$ is the resistance, $\rho$ is the conductivity, $L$ is the length, and $S$ is the cross-sectional area. The cross-sectional area is the product of the thickness of the lower LMGC conductive layer and linewidth, and the thickness of the LMGC conductive layer is calculated using Eq. (2).

2 $${t}_{conductive}=\frac{{V}_{LM}}{\phi \cdot S}$$

where ${t}_{conductive}$ is the thickness of the LMGC conductive layer, ${V}_{LM}$ is the volume of the added LM, $\phi$ is the filling factor of the LM in the LMGC conductive layer, and $S$ is the bottom surface area of the pattern. The filling factor $\phi$ was calculated by dividing the theoretical LMGC layer of 100% filling factor by the actual thickness, $\phi$ = 86.0%. Filling factor measurements were conducted by measuring actual LMGC layer thickness using scanning electron microscopy-energy dispersive X-ray spectroscopy (SEM EDX). These measurements were performed only for wiring of 1.54 mm linewidth. Because volume ratios of ionic liquid, polymer, solvent, and LM are constant for all linewidth wirings, ${t}_{conductive}$ can be calculated from the amount of LM added using Eq. (2). To define the theoretical curve, the conductivity of the ion gel–LM bilayer wiring was optimized to minimize the average difference between the measured and theoretical resistance values. In this case, the conductivity was $\rho =1.58\times {10}^{6} \mathrm{S}/\mathrm{m}$. As shown in Fig. 2b, smaller linewidths of the ion gel–LM bilayer wiring result in larger measured resistances. Furthermore, the theoretical and measured values are almost in agreement. Supplementary Fig. S3 shows the theoretical value when the conductivity of Galinstan ($\rho =3.46\times {10}^{6} \mathrm{S}/\mathrm{m}$)^11^ is used.

Supplementary Fig. S4 shows the relationship between the thickness of the LMGC conductive layer and resistance. The theoretical curve in Supplementary Fig. S4 is identical to that shown in Fig. 2b; thinner LMGC conductive layers result in higher resistances. In addition, thicker LMGC conductive layers result in measured resistances that are significantly larger than the corresponding theoretical values. Supplementary Fig. S5 shows the change in resistance when the ionic liquid was changed from *N*-methyl-*N*-propylpyrrolidinium bis(fluorosulfonyl)imide (IL 1) to *N*,*N*,*N*-trimethyl-*N*-propylammonium bis(trifluoromethanesulfonyl)imide (IL 2), 1-ethyl-3-methylimidazolium tetrafluoroborate (IL 3), 1-ethyl-3-methylimidazolium bis(trifluoromethanesulfonyl)imide (IL 4), and trihexyl(tetradecyl)phosphonium bis(trifluoromethylsulfonyl)imide (IL 5) with the same wiring shape. The maximum difference between each measured value and the average value was 5.73%. Supplementary Fig. S6 shows the relationship between the thickness of the ion gel insulating layer and resistance of the ion gel along the thickness direction. The thickness of the ion gel insulating layer is calculated using Eq. (3).

3 $${t}_{insulative}=\frac{{m}_{IG} }{{S  \cdot d}_{IG}}$$

where ${t}_{insulative}$ is the thickness of the ion gel insulating layer, ${m}_{IG}$ is the mass of the added ion gel, $S$ is the bottom surface area of the pattern, and ${d}_{IG}$ is the density of the ion gel. The density of the ion gel is calculated by dividing the theoretical mass per unit bottom surface area of the added ion gel by the actual thickness of the ion gel insulating layer measured using SEM EDX, ${d}_{IG}=2.47 \mathrm{g}/\mathrm{mL}$. In Supplementary Fig. S6, the resistance decreases proportionally as the ion gel insulating layer becomes thinner, and a sharp decrease in resistance is observed at a thickness of 82.7 μm. This indicates that the ion gel layer did not function as an insulative layer at a thickness of 82.7 μm due to mechanical instability of the used ion gel. Furthermore, the ion gel–LM bilayer wiring became extremely fragile and difficult to fabricate when the thickness of the ion gel insulating layer was less than 82.7 μm. The correlation coefficient between the thickness and resistance of the ion gel insulating layer is 0.987.

Figure 2c shows the relationship between the particle diameter of the LM when casting the solution and conductivity. As shown in Fig. 2c, smaller LM particle sizes result in lower conductivity. In addition, the graph with the logarithm of the LM particle diameter as the abscissa shows that the conductivity and LM particle diameter have a linear relationship with a correlation coefficient of 0.949. Figure 2d and e show the results of the SEM EDX analysis of the cross-sections of the flexible materials comprising LM mixed with PDMS (PDMS–LM composite) and LM mixed with ion gel (ion gel–LM bilayer). In both cases, the LM particle diameter during casting was adjusted to approximately 150–200 µm. Figure 2d shows that in the case of the PDMS–LM composite, the LM mixed with PDMS is relatively evenly dispersed in PDMS, the PDMS walls that separate the LM particles are thick, and a PDMS film is formed on the bottom surface. In contrast, Fig. 2e shows that in the case of the ion gel–LM bilayer, the LM mixed with the ion gel is concentrated at the bottom to form particles with large diameters, the ion gel walls that separate the particles are thin, and the LM is exposed from the bottom surface. The LM deposited and exposed at the bottom serves as the conductive layer and the thick ion gel remaining at the top serves as the insulative layer. Figure 2e shows that the thickness of the ion gel–LM bilayer is approximately 400 μm. In addition, the ion gel partially infiltrates the space between the LM particles, and the high surface tension of the LM prevents it from seeping out while maintaining the structure of this composite. Supplementary Fig. S7 shows the results of tensile tests using ion gel–LM bilayer wiring. The ion gel–LM bilayer wiring withstands strain up to 100%, and the resistance change is − 44.8%. In addition, the resistance of the ion gel–LM bilayer wiring decreases gradually after a large decrease when 50% strain is applied repeatedly for 100 cycles. As shown in Supplementary Fig. S8, the ion gel–LM bilayer wiring and curved surface with a radius of curvature of 1 mm make conformal contact, and the resistance of the ion gel–LM bilayer wiring is 46.8 mΩ. As shown in Fig. 2f, the ion gel–LM bilayer wiring can be applied to other flexible substrates, such as PDMS, Ecoflex, PET film, and acrylamide hydrogel, indicating that the method in this study can be applied to various flexible substrates.

### Verification of insulative layer performance

**Figure 3:**
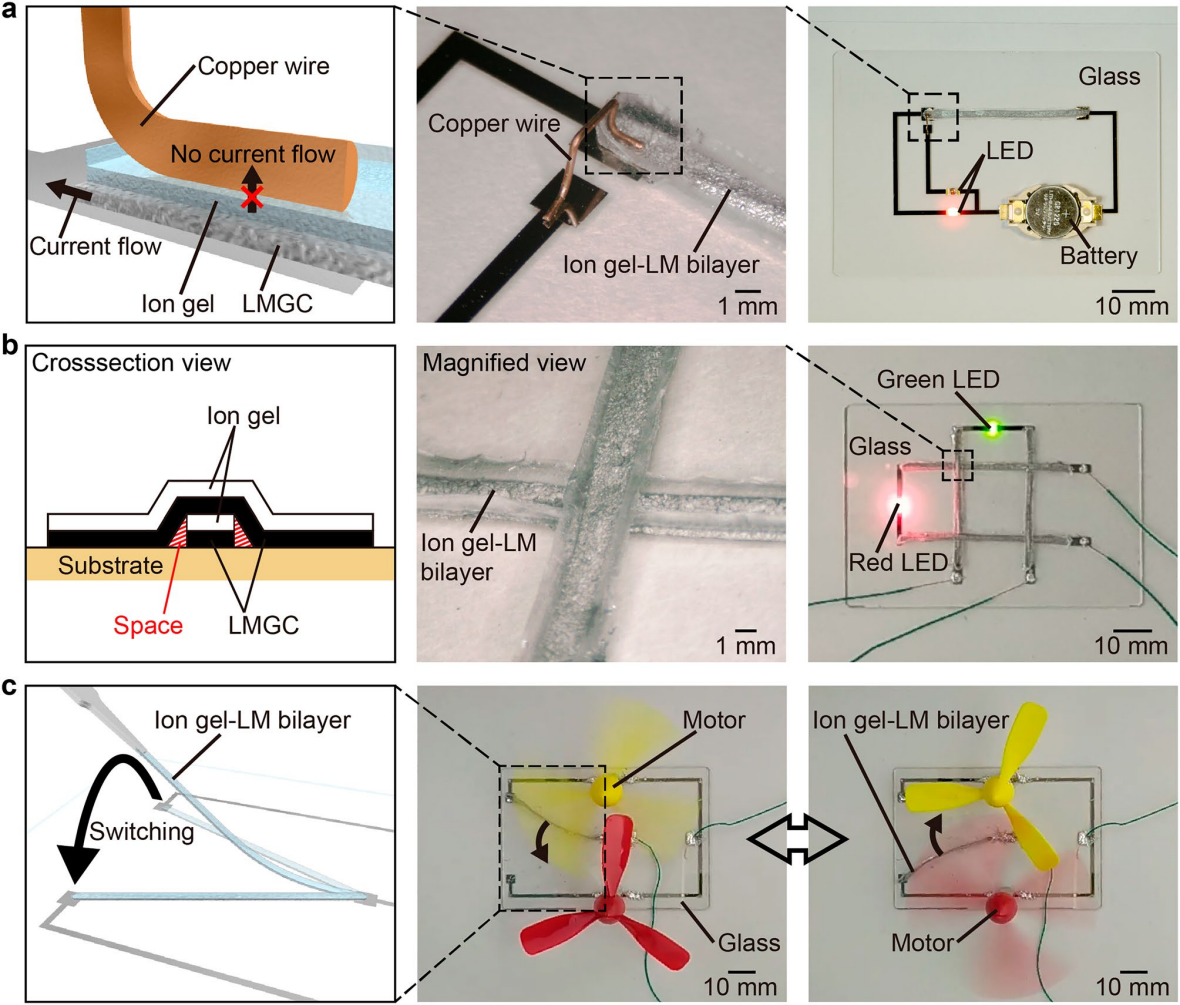
Demonstrations using the ion gel–LM bilayer wiring. (**a**) Verification of the insulative layer performance of the ion gel–LM bilayer wiring. The light-emitting diode (LED) connected to the bottom LMGC conductive layer turned on, but the LED connected to the top ion gel insulating layer did not. (**b**) Construction of a 3D mesh structure using ion gel–LM bilayer wiring. (**c**) Switching circuit using the ion gel–LM bilayer wiring.

As shown in Fig. 3a, the ion gel–LM bilayer wiring in this study serves as an adequate insulative layer. The ion gel–LM bilayer wiring has a copper wire on the top surface and an Ni electrode on the bottom surface, each of which was connected to a light-emitting diode (LED). In other words, each of the insulative and conductive layers of the ion gel–LM bilayer wiring was physically connected to the LED. On connecting a battery to this circuit, the lower LED connected to the conductive layer turned on, whereas the upper LED connected to the insulative layer did not. This result indicates that only the conductive layer of the ion gel–LM bilayer wiring was effectively electrically connected and served as the circuit and that the insulative layer properly executed its expected function.

### 3D wiring

As shown in Fig. 3b, 3Dwiring was performed using ion gel–LM bilayer wiring. Supplementary Video S1 shows the experimental results. As shown in the cross-sectional view in Fig. 3b, the four-ion gel–LM bilayer wirings were arranged orthogonally, and their tops and bottoms were swapped at the crossing points to construct the mesh structure. The structure, where two closed circuits intersected, was fabricated by connecting two LEDs and power supplies. The four-ion gel–LM bilayer wirings constructed as mesh structures were electrically independent because of the self-assembled ion gel insulating layer on the top. Therefore, the two LEDs connected by the self-assembled LMGC conductive layer at the bottom can be turned on independently (Supplementary Fig. S9). In addition, no electrical crosstalk occurs even in a bent state (Supplementary Fig. S10).

### Switching

Another advantage of the ion gel–LM bilayer wiring used in this study is that it is easy to move. The results are shown in Fig. 3c. The ion gel–LM bilayer wiring was lifted with tweezers and moved between the electrodes to be used as a switch in the circuit. As shown in Fig. 3c, the upper and lower motors were switched on and off as desired (Supplementary Video S2). No LM dripped from the ion gel–LM bilayer wiring when moving. In addition, as shown in Supplementary Fig. S11, the ion gel–LM bilayer wiring adhered well to the substrate before lifting with tweezers and did not peel off from the substrate, even when tilted up to 180°. The connection with the electrode in contact with the conductive layer was also stable with a resistance change of ± 1.36% (Supplementary Fig. S11). As shown in Supplementary Fig. S12, after the glass plate was attached to the force gauge and pressed against the fixed ion gel–LM bilayer wiring so that the glass plate contacted the entire bottom surface of the wiring, the peel force was obtained as the maximum tensile force when the force gauge was pulled up. Supplementary Fig. S13 shows the relationship between time and measured tensile force when using the ion gel–LM bilayer wiring with a width of 2 mm. The peel force of the ion gel–LM bilayer wiring with a width of 2 mm from glass is $117\times {10}^{-3}$ N, which was greater than the gravitational force ($F=1.85\times {10}^{-3} \mathrm{N})$. Therefore, the ion gel–LM bilayer wiring did not peel off naturally from the glass substrate even when tilted 180°.

### Optical sensing circuit

**Figure 4:**
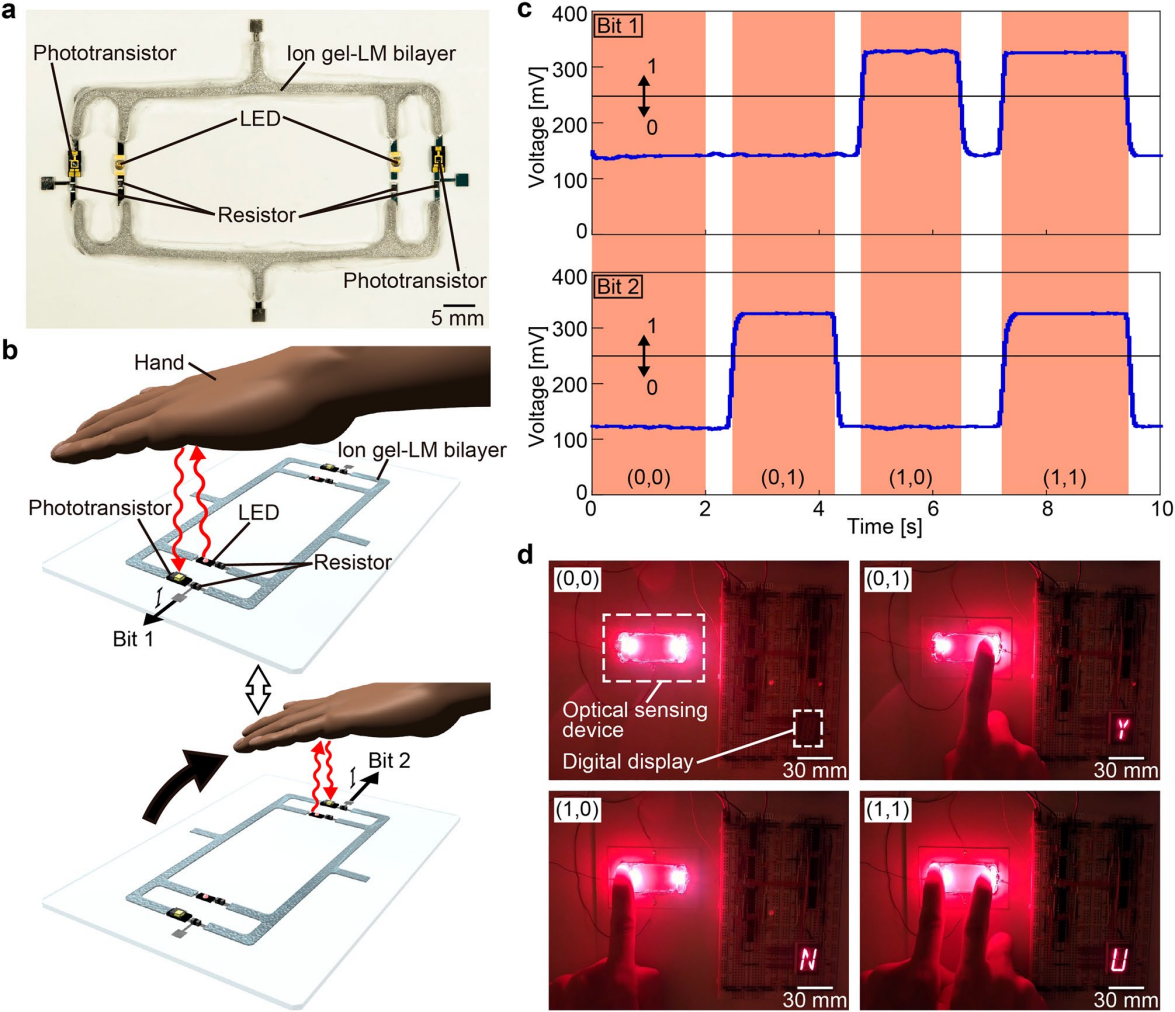
Optical sensing circuit using ion gel–LM bilayer wiring. (**a**) Top view of the constructed circuit. (**b**) Schematic of the operation of the circuit. On placing a hand over the LED, the light from the LED is reflected and received by the phototransistor, causing an electric current to flow. (**c**) Relationship between time and voltage value of 2-bit outputs obtained from the circuit. The voltage value increases as the hand is brought closer. (**d**) Four-character patterns controlled by hand movements using the external device connected to the circuit.

The ion gel–LM bilayer wiring in this study is suitable for use as circuit wiring because of its high and stable conductivity that can be obtained without any activation process. Figure 4a shows the optical sensing circuit fabricated using the ion gel–LM bilayer wiring. As shown in Fig. 4b, on placing a hand over the LED, the light from the LED is reflected and received by the phototransistor, causing an electric current to flow. By connecting two of these circuits symmetrically in parallel, a 2-bit output is obtained. Figure 4c shows the voltage change of the 2-bit output; the voltage value is high when the hand is brought close to the LED. As shown in Fig. 4c, the analog signal through the ion gel–LM bilayer wiring is stably outputted. In addition, as shown in Fig. 4d, the analog signal is converted to a digital signal by setting the threshold voltage value and connected to the external device to display four-character patterns on the eight-segment display. When the hand is not placed on the circuit, nothing is displayed; when the hand is placed on the right side of the circuit, “Y” is displayed; when the hand is placed on the left side of the circuit, “N” is displayed; and when the hand is placed on both the left and right sides of the circuit, “U” is displayed (Supplementary Video S3). In addition, the circuit operates properly even in a bent state (Supplementary Fig. S14). Thus, the fabricated ion gel–LM bilayer wiring can be used for circuit wiring.

## Discussion

As shown in Fig. 2a, the linewidth of the ion gel–LM bilayer wiring is uneven in some areas. This uneven linewidth of the ion gel–LM bilayer wiring can be attributed to the fact that the dispersion of LM is not completely uniform after casting. When the distribution of the ion gel and LM was not uniform, the thickness ratio of the ion gel and LMGC layers was also not uniform, and the part with a thin LMGC layer had a relatively thicker ion gel layer than the part with a thicker LMGC layer. The solvent volatilized only from the top surface; thus, the thicker ion gel layer resulted in slower gelation, which leads to larger shrinkage and finer linewidths. A smaller LM particle size and higher solution viscosity can improve the uniformity of LM dispersion; however, obtaining high conductivity without sintering processes becomes difficult. Figure 2b and e show that the minimum linewidth of the ion gel–LM bilayer wiring in this study is 404 μm, and the thickness is approximately 400 μm, including the insulative layer. In a previous study that enabled LM wiring on gel substrates, a minimum linewidth of approximately 500 μm and a thickness of approximately 10 μm were achieved by wiring LM mixed with Ni microparticles using a magnet^24^. Furthermore, by using a mask during wiring in the same manner, the thickness increased to approximately 56 μm, but the minimum linewidth was reduced to 85 μm^39^. In another study, the sintering of hydrogels mixed with LM particles was used to achieve a linewidth of approximately 3.3 μm^29^, while the transfer of LM onto a hydrogel with high wettability with LM has achieved a linewidth of approximately 100 μm^40^. Compared to these methods, the ion gel–LM bilayer wiring in this study has a larger linewidth and thickness. However, the wiring and encapsulation of the ion gel–LM bilayer occur simultaneously owing to the self-assembly of the ion gel at the top. Furthermore, the bilayer can be applied to various substrates after wiring, as shown in Fig. 2f.

Wiring and encapsulation can also be integrated using injection or PDMS–LM composites. In particular, LM wiring with a width of 70 μm and thickness of 70 μm can be fabricated by injection wiring^15^, and arbitrary shapes can be fabricated with a three-axis stage by wiring using a PDMS–LM composite^35^. In contrast, the method used in this study enables easy patterning of branched shapes, as shown in Fig. 4a. Furthermore, because parts of the LM exposed from the bottom connect to each other naturally, the ion gel–LM bilayer does not depend on the percolation network, which is characteristic of LM–polymer composites^37^, and can achieve high and stable electrical conductivity without activation processes such as mechanical sintering. The parts of the LM are exposed from the bottom of the ion gel–LM bilayer wiring fabricated in this study and naturally connected to each other as confirmed by the SEM EDX images shown in Supplementary Fig. S15.

The current minimum linewidth depends on the patterning method, and a smaller linewidth can be achieved by casting a smaller amount of solution uniformly into the mold. To achieve uniform casting, the solvent must not volatilize quickly, and the viscosity of the solution must be low. Therefore, the minimum linewidth of ion gel–LM bilayer wiring can be further decreased by optimizing the polymer, ionic liquid, and solvent. Furthermore, the interval distance between the wires might be decreased by using a metal mold to increase adhesion to the substrate with a magnet.

Figure 2b shows that the measured resistance values for different linewidths are almost in agreement with the theoretical values, which indicates that the conductivity of the ion gel–LM bilayer wiring is optimal. This high conductivity can be attributed to the fact that the LM is exposed at the bottom of the ion gel–LM bilayer and that the contact resistance between the LM particles is low because the LM particles are electrically connected despite the infiltration of the ion gel. Supplementary Fig. S3 shows that the optimized conductivity of the ion gel–LM bilayer wiring is less than that of Galinstan, which can be attributed to the partial infiltration of the ion gel between the LM particles. From Supplementary Fig. S4, as the LMGC conductive layer thickens, the difference between the measured and theoretical values of resistance increases, indicating that the conductivity of the ion gel–LM bilayer wiring decreases. The LMGC conductive layer was composed of two parts: the LM part exposed from the ion gel and the LM part mixed with the ion gel. Therefore, this decrease in conductivity can be attributed to the fact that the thickness of the LM part exposed from the ion gel, which has a higher conductivity than the LM part mixed with the ion gel, is difficult to change when the thickness of the LMGC conductive layer changes.

Depending on the intended application, several studies have investigated the combined use of ion gels and conductive materials. Thin-film transistors that exhibit low-voltage operation and high device performance can be realized using an ion gel as a high-capacitance gate insulator and Au as a conductive material^41^. Capacitive pressure sensors with high sensitivity can be realized using ion gels as electrodes with high ionic conductivity and carbon nanotubes (CNTs) as a conductive material^42^. Actuators with a long life that can operate in air at low voltage and high speed can be realized using an ion gel as an electrolyte layer and a bucky gel (a gelatinous ionic liquid containing CNTs) as a conductive material^43^. Flexible transparent electrodes with a high conductivity of approximately $2.0\times {10}^{6}$ S/m can be realized using ion gel as a protective layer and Ag nanowires as a conductive material^44^. Flexible and stretchable micro-supercapacitors can be realized using an ion gel as a dielectric and LM mixed with Cu as a conductive material^45^. In this study, the conductive material was patterned simply by casting it into a PDMS mold using an ion gel as an insulative layer and LM as a conductive material. This method enables easy patterning and high conductivity, owing to the sedimentation and contact of the LM at the bottom of the ion gel. Therefore, this method can serve as a flexible wiring method that does not require activation processes such as encapsulation and sintering.

Supplementary Fig. S5 shows that the maximum difference between the measured and average resistance values when the ionic liquid was changed is 5.73%, i.e., ∆R = 2.89 mΩ. This small change in resistance indicates that the ionic liquid does not change the resistance of the ion gel–LM bilayer. Supplementary Fig. S6 shows that the insulating performance deteriorates when the thickness of the ion gel insulating layer reaches 82.7 μm. A sharp decrease in resistance was observed even when a force below the measurement resolution of the force gauge used in this study (0.001 N) was applied to the ion gel insulating layer with a thickness of 82.7 μm. This indicates that the critical thickness of the ion gel insulating layer does not practically function as an insulating layer, although it may depend on the applied pressure. In addition, the ion gel–LM bilayer becomes extremely fragile when the thickness of the ion gel insulating layer decreases below 82.7 μm. However, the bilayer can be made more durable by selecting mechanically tough ion gels^46,47^ for thinning. Regarding the insulating properties, the regression line in Supplementary Fig. S6 shows that the conductivity of the ion gel insulating layer is $2.02\times {10}^{-6}$ S/m, which is sufficient to obtain an insulative resistance of 0.1 MΩ at a layer thickness of 16.8 μm.

Figure 2c shows that the conductivity decreases as the LM particle diameter decreases. This decrease in conductivity can be attributed to the easier infiltration of ion gel between the LM particles and the larger amount of oxide layer on the LM as the LM particle diameter decreases. The particle diameter of the LM must be minimized to decrease the linewidth of ion gel–LM bilayer wiring. However, ensuring adequate conductivity, and improving it, when the particle diameter of the LM is decreased is a future issue.

Figure 2d and e show that the ion gel–LM bilayer has thinner partition walls separating the LM particles than the PDMS–LM composite. This reduced thickness can be attributed to the lower viscosity and specific gravity of the ion gel compared to PDMS because of the presence of a large amount of MEK as a solvent in the ion gel in the pre-cured state. Consequently, the mixed LM particles are more easily sedimented and the interparticle distance is reduced, which could also explain why parts of the LM are exposed from the bottom of the ion gel–LM bilayer. Even polymers such as PDMS can be made to have lower viscosity and specific gravity using solvents such as tetrahydrofuran (THF). However, the resulting solution is highly reactive, limiting the molds and substrates that can be used.

As shown in Supplementary Fig. S7, the resistance of the ion gel–LM bilayer wiring decreases when stretched, which can be attributed to the breaking down of the ion gel wall separating the LM particles or oxide skin of the LM. In addition, the resistance of the ion gel–LM bilayer wiring decreases gradually after a large decrease when 50% strain is applied repeatedly for 100 cycles, which can be attributed to the large initial sintering followed by gradual additional sintering. This indicates that sintering is effective to further improve the conductivity. Plastic deformation causes irreversible changes in the wiring shape. Therefore, for future applications in wearable and stretchable devices, the elastic deformation range requires extension, which may be achieved by changing the ratio and type of ionic liquid and polymer.

Furthermore, the proposed ion gel–LM bilayer enables easy 3D wiring because of the top insulative layer, as shown in Fig. 3b. Conventionally, approaches such as introducing LM into 3D channels^20,33,48^, stacking 2D LM wiring^49^, fabricating freestanding LM using a dispenser^25,50,51^, and fabricating 3D structures of LM using a self-healing hydrogel as a support^52^ have been used to fabricate 3D LM wiring. Introducing LM into 3D channels involves easy construction and high-accuracy single-stroke patterning, whereas the method of stacking 2D LM wiring can fabricate more precise fine lines using a soft lithography process. Similarly, fabricating freestanding LM using a dispenser enables ultra-fine lines to be drawn with high spatial flexibility. Finally, fabricating 3D structures of LM using a self-healing hydrogel as a support enables the easy fabrication of 3D structures on a larger scale. In contrast to these methods, the ion gel–LM bilayer wiring used in this study results in a minimum linewidth of 404 μm and a thickness of approximately 400 μm, including the insulative layer, which presents a challenge when attempting to improve the accuracy. However, ion gel–LM bilayer wiring involves easy patterning, even in the case of branched shapes, and can be applied to arbitrary substrates. Furthermore, the fabricated electrodes are supported by an ion gel, which is stronger than the LM itself and provides excellent shape retention in 3D space. The ion gel–LM bilayer wiring in this study was not insulated on the sides. However, as shown in the cross-sectional view in Fig. 3b, the ion gel–LM bilayer wirings are electrically independent on the sides when stacked due to the space between the upper and lower wirings. As a future prospect, the LM wiring may be fully covered by fabricating a mold with a microstructure that can be infiltrated only by the ion gel.

As shown in Fig. 3c, the ion gel–LM bilayer wiring in this study can be lifted and moved with tweezers after wiring, which can be attributed to the fact that the ion gel held the LM in place (Fig. 2e). In similar methods that enable wiring on arbitrary substrates, the transfer of patterns of Au thin film^53^, Ag nanoparticles^54^, and LM^19^ fabricated on polyvinyl alcohol (PVA) film have been reported. These methods enable fine line drawing and are effective for integration and systemization. In contrast, as shown in Figs. 1e and 3c, the ion gel–LM bilayer wiring can be moved to an arbitrary location by lifting it with tweezers even after wiring, which enables easy fine positioning and reuse of the wiring, in contrast to conventional methods. Owing to the infiltration of the ion gel between the LM particles and the high surface tension of the LM, the structure of the composite was maintained without LM seeping out. Therefore, the LM may seep out of the wiring and remain on the substrate side when the LM particles become larger or when the LMGC layer thickens. Supplementary Fig. S16 shows the adhesion of LM when the ion gel–LM bilayer wiring with an LM particle size of 167 μm and LMGC layer thickness of 0.260 mm was applied to glass, polyimide film, Ni electrode, Au electrode, PDMS, and fluorine-coated PDMS. As shown in Supplementary Fig. S16, when the LM particle size is 167 μm and the thickness of the LMGC layer is 0.260 mm, the LM adheres only to the Au electrode and PDMS. In contrast, no LM adhered to glass, polyimide film, Ni electrodes, or fluorine-coated PDMS even when the LM particle size increased from 167 to 1112 μm and thickness of the LMGC layer was increased from 0.260 to 1.04 mm. This indicates that the substrate material was the dominant factor that caused the LM to seep out of the ion gel–LM bilayer wiring and remain on the substrate side. The adhesion of LM to the substrate may interfere with the use of ion gel–LM bilayer wiring for switching. As shown in Supplementary Fig. S17, no LM drips when the thickness of the LMGC layer changed from 0.260 to 1.04 mm. This indicates that the gravitational force on the LM was smaller than its surface tension and force of the ion gel infiltrating between the LM particles and holding the LM in place.

Furthermore, the ion gel–LM bilayer wiring, exhibits high and stable conductivity and is suitable for fabricating electronic circuits, such as the optical sensing circuit in this study, as shown in Fig. 4a,. The circuit was constructed on a glass substrate because it was necessary to fabricate electrodes. However, as shown in Fig. 2f, the wiring can be applied on the gel. Moreover, circuits can be fabricated using only low-Young 's modulus materials such as gels. In addition to LM, conductive gels using CNT^55,56^, PEDOT:PSS^57^, and polyaniline^58^ have been reported as conductive materials with a Young's modulus as low as that of the gel. These conductive materials are useful as sensor elements, such as pressure and strain sensors, because they often use the percolation effect and have a large resistance change with strain. In contrast, the conductivity of the ion gel–LM bilayer wiring in this study is approximately $1.58\times {10}^{6}$ S/m, which is suitable for wiring in circuit construction because of its high conductivity and low resistance change rate without depending on the percolation effect. Furthermore, the ion gel–LM bilayer wiring can be easily moved after wiring and can potentially be applied directly to human skin for health monitoring.

Supplementary Table S1 presents the results of a comparison between the LM wiring technology in this study and conventional LM wiring technologies. Although the LM wiring technology in this study is inferior to conventional technologies in terms of resolution and requires improvement, it has notable advantages in terms of encapsulation, design flexibility, and conductivity compared to conventional technologies.

In summary, this study proposed a method to realize LM wiring without additional activation processes while using ion gel to obtain a self-assembling insulative layer on top of the LM wiring. The solution, prepared by mixing LM, ionic liquid, and polymer in a solvent, was patterned on a substrate using a casting method. The LM sedimented before the ion gel was cured, resulting in a self-assembled bilayer structure with a bottom conductive layer of the LMGC and a top insulative layer of the ion gel. The LM particles formed during mixing came into contact after sedimentation without any activation process owing to the physical properties of the ion gel, resulting in high conductivity and low resistivity change. Furthermore, the ion gel–LM bilayer wiring can be easily moved with tweezers because the ion gel partially infiltrated the space between the LM particles, and the high surface tension of the LM itself prevented the LM from seeping out while maintaining the bilayer structure. Although the ion gel–LM bilayer wiring requires improvement with a minimum linewidth of 404 μm, it can be applied to gels as well as conventional substrates such as PDMS and Ecoflex. In addition, it can be stacked to fabricate 3D wiring, and the ion gel insulating layer prevents electrical crosstalk between multiple wirings. It can also be used for switching by lifting and moving it between the electrodes, allowing the circuit to be turned on and off as desired. Furthermore, ion gel–LM bilayer wiring can be used to fabricate the optical sensing circuit, for which stable operation was confirmed from the output analog signal. Finally, the proposed method does not require sintering and encapsulation, thus simplifying circuit fabrication technology by printing. In addition, the flexibility of the component materials makes the proposed wiring suitable for wearable devices, implantable devices, and gel actuators that can measure the health status of the human body by applying ion gel–LM bilayer wiring directly to biological tissues.

## Materials and methods

### Materials and reagents

Galinstan was obtained from E-Material, Ltd. (Tokyo, Japan). PVDF-HFP was obtained from Arkema (Colombes, France). PVA, MEK, and trihexyl(tetradecyl)phosphonium bis(trifluoromethylsulfonyl)imide were obtained from Wako Pure Chemical Industries Ltd. (Osaka, Japan). *N*-methyl-*N*-propylpyrrolidinium bis(fluorosulfonyl)imide and *N*,*N*,*N*-trimethyl-*N*-propylammonium bis(trifluoromethanesulfonyl)imide were obtained from Kanto Chemical Co., Inc. (Tokyo, Japan). 1-Ethyl-3-methylimidazolium tetrafluoroborate and 1-ethyl-3-methylimidazolium bis(trifluoromethanesulfonyl)imide were obtained from Tokyo Chemical Industry Co. Ltd. (Tokyo, Japan). PDMS (Sylgard 184) was obtained from Dow, Inc. (Michigan, US).

### Fabrication method of ion gel–LM bilayer

Figure 1c shows the fabrication method of the ion gel–LM bilayer. The PVDF-HFP polymer and MEK solvent were mixed in a 4 mL vial at a ratio of 1:21 (wt%) and stirred for 15 min to dissolve the polymer. *N*-methyl-*N*-propylpyrrolidinium bis(fluorosulfonyl)imide, an ionic liquid, was added at a ratio of ionic liquid: polymer = 7:3 (wt%) and stirred for 5 min for dissolution. LM was added to the solution at a concentration of 65 vol% and stirred at a controlled rotational speed and time to allow it to reach an arbitrary particle size in the solution. The solution was cast into a PVA-coated PDMS mold attached to a surface-treated glass substrate, and LM particles were uniformly dispersed using a vortex mixer (SI-0286; Scientific Industries, Inc., New York, US) and left at room temperature for 1 h. After the LM was sedimented and the ion gel was cured, the PDMS mold was peeled off from the glass substrate and composite gel by dripping water heated to 100 °C onto it to complete the fabrication of the ion gel–LM bilayer.

### Fabrication method of PDMS mold

In this study, PDMS was selected as the mold material because the mold for casting must be durable against the MEK solvent and adhere strongly to the substrate to prevent leakage of the solution. The PDMS mold was fabricated by pouring PDMS into a polyacetal resin (POM) mold cut using a subtractive rapid prototyping machine (MDX-540; Roland DG Corporation, Shizuoka, Japan) and curing in the mold.

### Method of coating PVA to PDMS mold

The PVA sacrificial layer was coated on the inner side of the PDMS mold to simplify the process of peeling the ion gel off from the PDMS mold. The PVA used in this study was a fully saponified type with an average degree of polymerization of approximately 1500–1800. An aqueous PVA solution was prepared by mixing 10 wt% PVA in pure water, heating, and stirring at 200 °C and 1500 rpm to dissolve the PVA, followed by lowering the solution temperature to 120 °C. The aqueous PVA solution was applied to the inside of the PDMS mold and left in an oven at 70 °C for 30 min to obtain a PVA-coated PDMS mold.

### Fabrication method of PDMS–LM composite

For comparison with the ion gel–LM bilayer, a PDMS–LM composite was fabricated, as shown in Supplementary Fig. S18. LM (90 wt%) was added to PDMS and mixed using a vortex mixer to form LM particles. The PDMS–LM composite was cast onto a substrate and left to cure at room temperature for 24 h.

### Measuring methods of linewidth and line thickness of the ion gel–LM bilayer wiring

The linewidth of the ion gel–LM bilayer wiring, defined as the width of the area where LM exists in the top view shown in Supplementary Fig. S1, was measured under a microscope. The line thickness of the ion gel–LM bilayer wiring and the thicknesses of the conductive LMGC and insulative ion gel layers were measured using SEM EDX. The line thickness of the ion gel–LM bilayer wiring is defined as the thickness from the bottom of the ion gel infiltrated between LM particles to the top of the ion gel insulating layer, the thickness of the conductive LMGC layer is defined as the thickness from the top to the bottom of the LM, and the thickness of the insulative ion gel layer is defined as the thickness from the top of the ion gel to the top of the LM in the cross-sectional view shown in Fig. 2e.

### Verification of insulative layer performance

Ni electrodes were fabricated on a glass substrate by vapor deposition, and red LEDs (XZM2ACR55W-3, SunLED Company Ltd., Minnesota, US) were mounted on the electrodes. The fabricated ion gel–LM bilayer wiring was placed to ensure that the bottom conductive layer was connected to the Ni electrode, and a copper wire was placed to connect the top insulative layer to the Ni electrode. In this manner, each of the insulative and conductive layers of the ion gel–LM bilayer wiring were physically connected to the LEDs. Supplementary Fig. S19 shows the circuit diagram. When a battery was connected to this circuit, the lower LED connected to the conductive layer turned on, whereas the upper LED connected to the insulative layer did not.

### 3D wiring

Ni electrodes were fabricated on a glass substrate by vapor deposition and copper electrodes were fabricated on a polyimide substrate by etching. Red LEDs (KPTD-1608SURCK, Kingbright Electronic Co., Ltd., Taiwan) and green LEDs (KPTD-1608CGCK, Kingbright Electronic Co., Ltd., Taiwan) were mounted on both electrodes. Four-ion gel–LM bilayer wirings were fabricated and arranged to form a mesh structure. Supplementary Fig. S19 shows the circuit diagram. 3D wiring was applied to light the LEDs independently.

### Switching

Ni electrodes were fabricated on a glass substrate by vapor deposition, and motors (8712; Artec Co., Ltd., Osaka, Japan) with propellers were mounted on the electrodes. After arranging the fabricated ion gel–LM bilayer wiring, the circuit was switched by lifting one end of the wiring with tweezers and moving it between the electrodes. A circuit diagram is shown in Supplementary Fig. S19.

### Measuring method of peel force

Supplementary Fig. S12 shows the method for measuring the peel force. The ion gel–LM bilayer wiring was turned over and fixed flat on the glass substrate. A glass plate was attached to the force gauge (ZTA-DPU-5N, IMADA Co., Ltd., Aichi, Japan), and the force gauge was pressed against the ion gel–LM bilayer wiring allowing the glass plate to be in contact with the entire bottom surface of the ion gel–LM bilayer wiring. The peel force was then obtained as the maximum tensile force when the force gauge was pulled up.

### Optical sensing circuit

Ni electrodes were fabricated on a glass substrate by vapor deposition and copper electrodes were fabricated on a polyimide substrate by etching. Red LEDs (XZM2ACR55W-3, SunLED Company Ltd., Minnesota, US) and phototransistors (TEMT6000X01, Vishay Intertechnology, Inc., Pennsylvania, US) were mounted on both electrodes. Fixed resistors of 51.1 kΩ were connected to the phototransistors, and 100 Ω to the LEDs. Supplementary Fig. S20 shows the circuit diagram of the optical sensing circuit and external device. On placing a hand over the LED, the light from the LED was reflected and received by the phototransistor, and the external circuit was activated by the resulting current, confirming that the sensing circuit was operating. The analog output of the voltage change during this operation was obtained using an oscilloscope (PicoScope 2205A; Pico Technology Ltd., Cambridgeshire, UK).

## Supplementary Information

Supplementary Video 1.

Supplementary Video 2.

Supplementary Video 3.

Supplementary Information 1.

## Data Availability

The datasets generated during and/or analysed during the current study are available from the corresponding author on reasonable request.
