# Supplementary material for: Self-assembling bilayer wiring with highly conductive liquid metal and insulative ion gel layers
Source: Sci Rep. 2023 Apr 12;13:5929. doi: 10.1038/s41598-023-32580-x (PMC10097700; doi:10.1038/s41598-023-32580-x)
Supplement: Supplementary file 4 — Supplementary Information 1. [file 41598_2023_32580_MOESM4_ESM.pdf]

Supplementary Information

Self-assembling bilayer wiring with highly conductive liquid metal and insulative ion gel layers

Koki Murakami[1], Yuji Isano[1], Juri Asada[2], Natsuka Usami[2], Yutaka Isoda[3], Tamami Takano[3], Ryosuke Matsuda[1], Kazuhide Ueno[2,4], Ohmi Fuchiwaki[1,3]* & Hiroki Ota[1,3]*

[1]Department of Mechanical Engineering, Yokohama National University, 79-5, Tokiwadai, Hodogaya-ku, Yokohama, Kanagawa 240-8501, Japan. [2]Department of Chemistry and Life Science, Yokohama National University, 79-5, Tokiwadai, Hodogaya-ku, Yokohama, Kanagawa 240-8501, Japan. [3]Graduate School of System Integration, Yokohama National University, 79-5, Tokiwadai, Hodogaya-ku, Yokohama, Kanagawa 240-8501, Japan. [4]Graduate School of Engineering, Yokohama National University, 79-5, Tokiwadai, Hodogaya-ku, Yokohama, Kanagawa, 240-8501, Japan. *email: fuchiwaki-ohmi-xk@ynu.ac.jp; ota-hiroki-xm@ynu.ac.jp

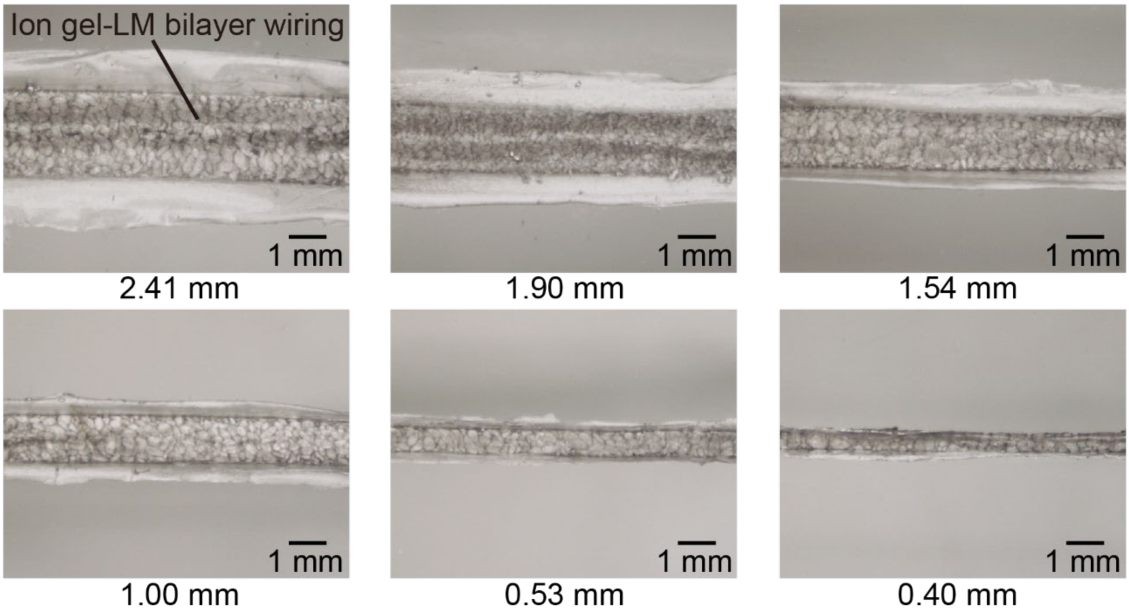

Supplementary Figure S1. Magnified view of the ion gel-liquid metal (LM) bilayer wiring. Linewidths are 0.40, 0.53, 1.00, 1.54, 1.90, and 2.41 mm.

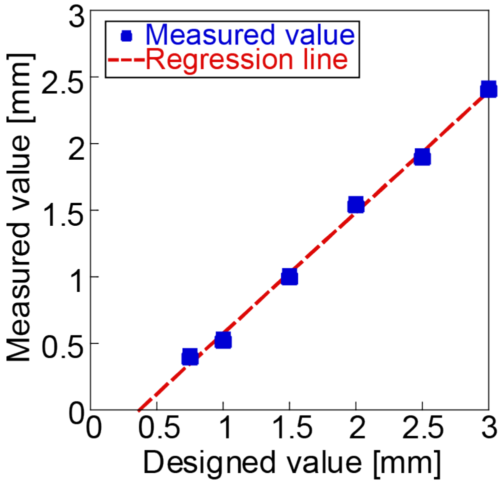

Supplementary Figure S2. Relationship between the linewidth designed by the polydimethylsiloxane (PDMS) mold and the actual measured linewidth of the ion gel-LM bilayer wiring. The measured values of the linewidth are, on average, 494 μm smaller than the designed values.

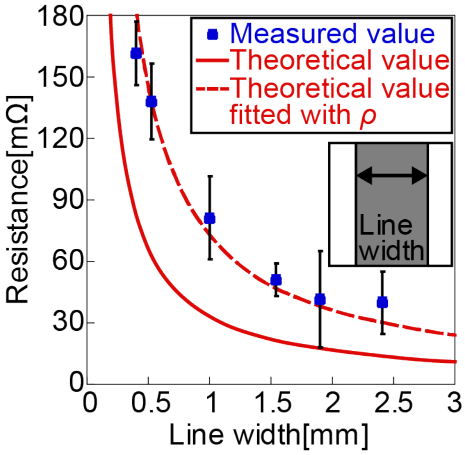

Supplementary Figure S3. Relationship between linewidths and measured resistances. Theoretical resistance values using Galinstan conductivity (solid line) and ion gel-LM bilayer wiring conductivity optimized from the experiment (dashed line).

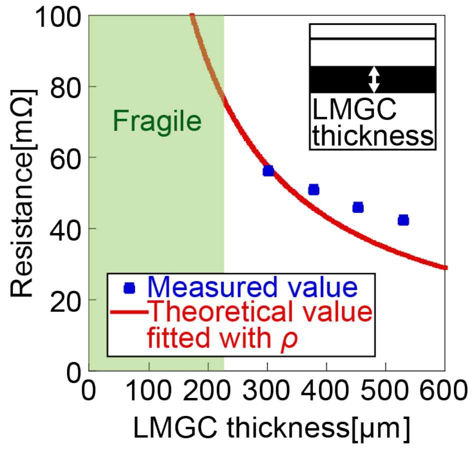

Supplementary Figure S4. Relationship between the thickness of the LMGC conductive layer and the resistance of ion gel-LM bilayer wiring. The resistance value decreases as a function of the LMGC thickness.

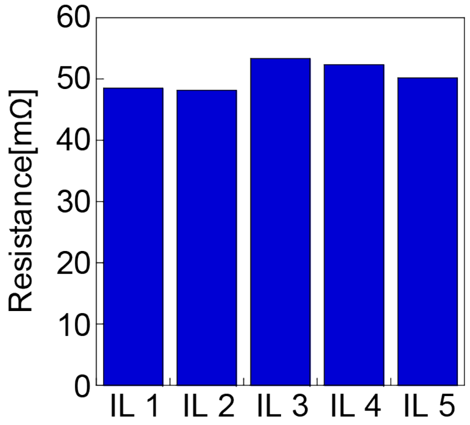

IL 1: N-Methyl-N-propylpyrrolidinium bis(fluorosulfonyl)imide
IL 2: N,N,N-Trimethyl-N-propylammonium bis(trifluoromethanesulfonyl)imide
IL 3: 1-Ethyl-3-methylimidazolium tetrafluoroborate
IL 4: 1-Ethyl-3-methylimidazolium bis(trifluoromethanesulfonyl)imide
IL 5: Trihexyl(tetradecyl)phosphonium bis(trifluoromethylsulfonyl)imide

Supplementary Figure S5. The resistance changes when the ionic liquid is changed from IL 1 to IL 2, IL 3, IL 4, and IL 5 with the same wiring shape.

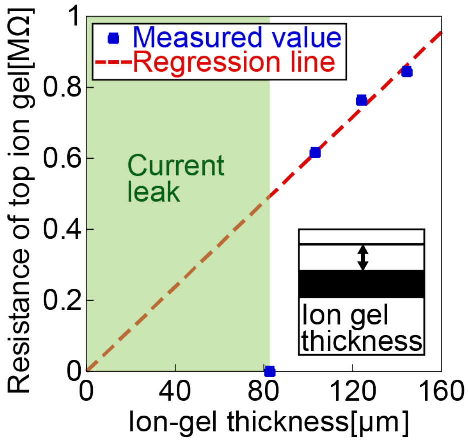

Supplementary Figure S6. Relationship between the thickness of the ion gel insulating layer and the resistance of the ion gel in the thickness direction of the ion gel-LM bilayer wiring.

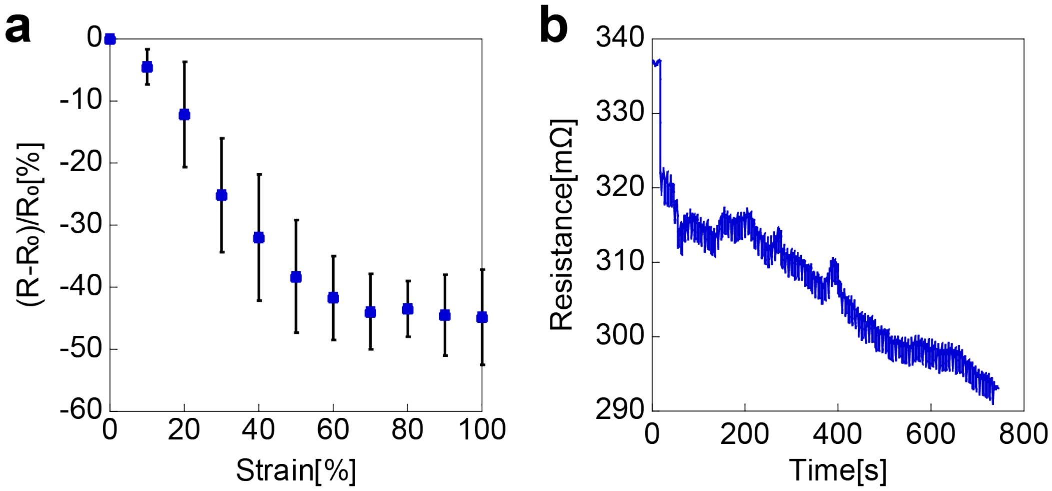

Supplementary Figure S7. Tensile test using ion gel-LM bilayer wiring. (a) Relationship between strain and resistance of ion gel-LM bilayer wiring. (b) Cyclic stretch-release test at 50% strain using ion gel-LM bilayer wiring.

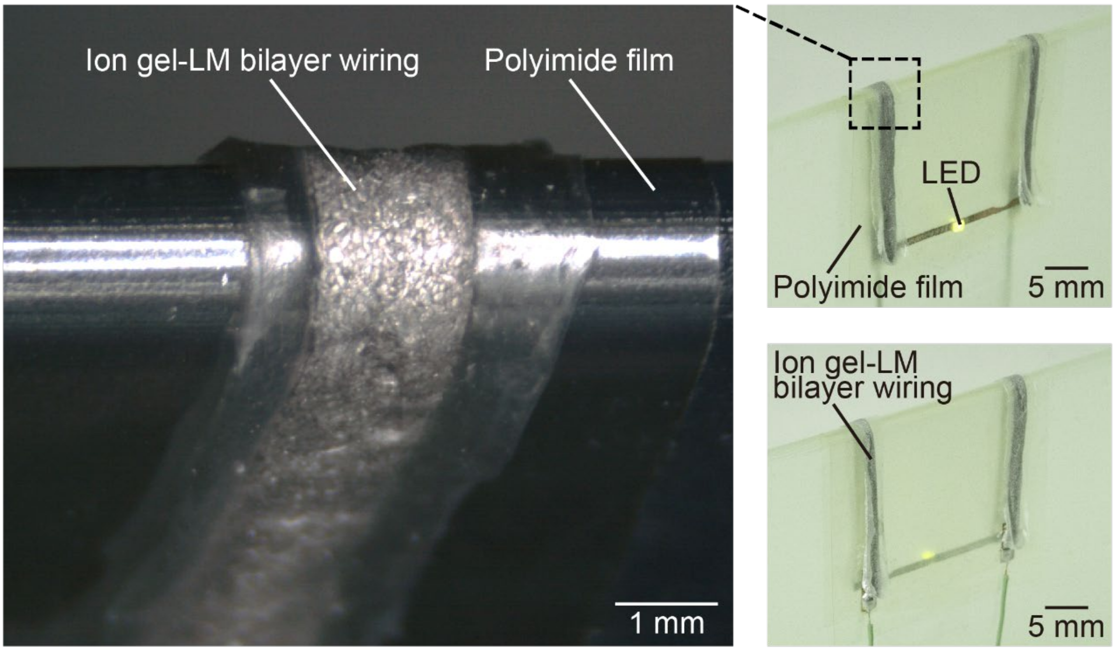

Supplementary Figure S8. Contact between the ion gel-LM bilayer wiring and curved surface with a radius of curvature of 1 mm.

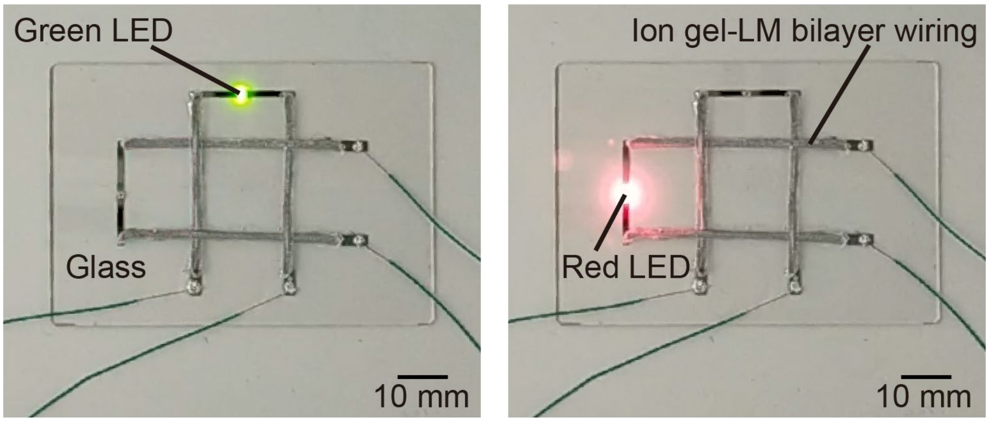

Supplementary Figure S9. Independent lighting of each light emitting diode (LED) in the three-dimensional structure of ion gel-LM bilayer wiring.

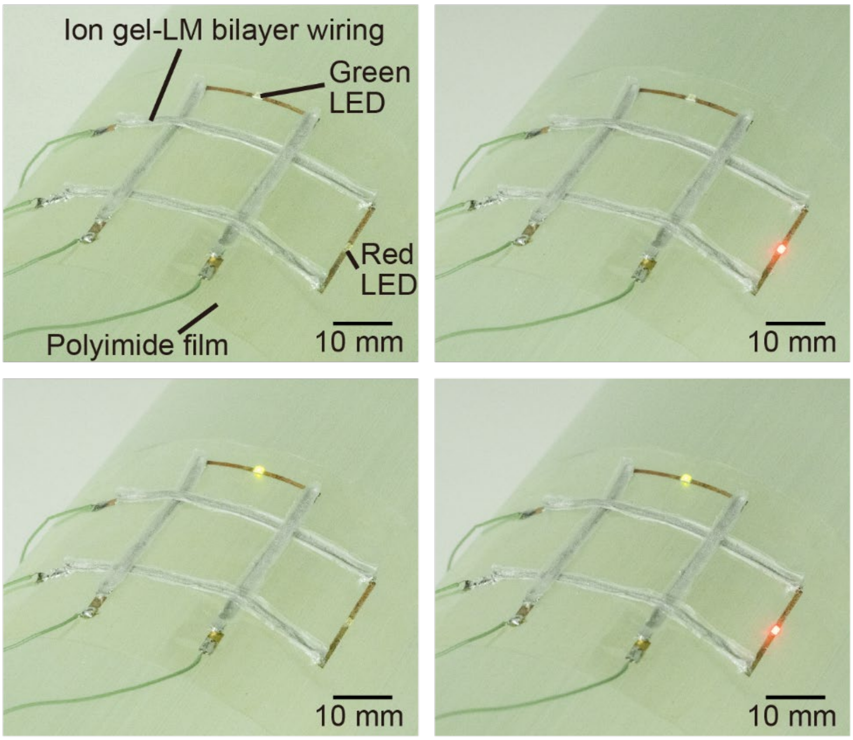

Supplementary Figure S10. Independent lighting of each LED when bending the three-dimensional structure of ion gel-LM bilayer wiring on the flexible substrate.

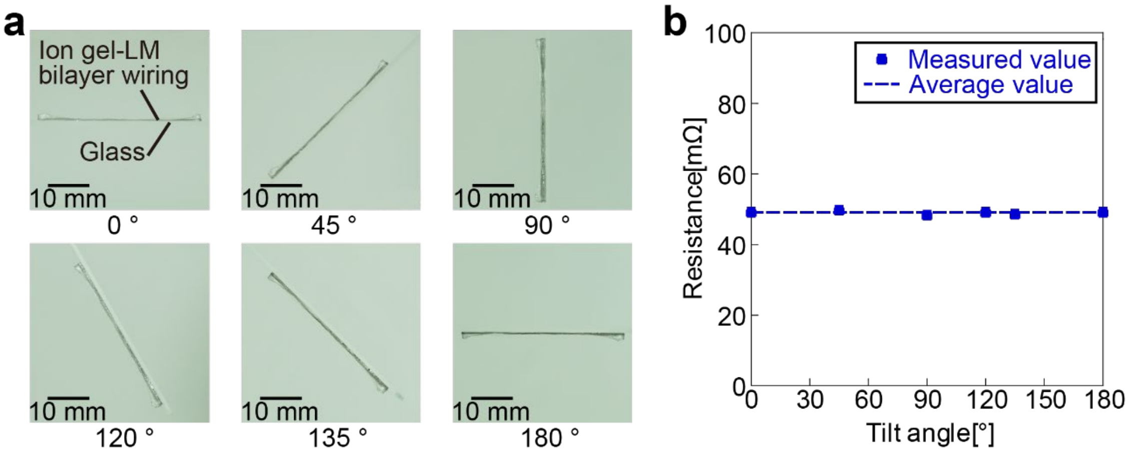

Supplementary Figure S11. Tilt experiment of ion gel-LM bilayer wiring. (a) Photographs of ion gel-LM bilayer wiring tilted from 0 ° to 180 °. (b) Relationship between tilt angle and measured resistance of ion gel-LM bilayer wiring.

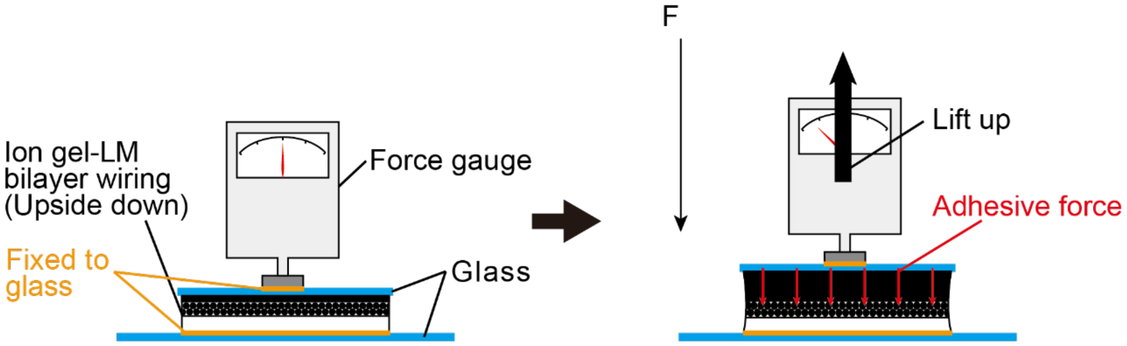

Supplementary Figure S12. Measuring method of peel force of ion gel-LM bilayer wiring from glass.

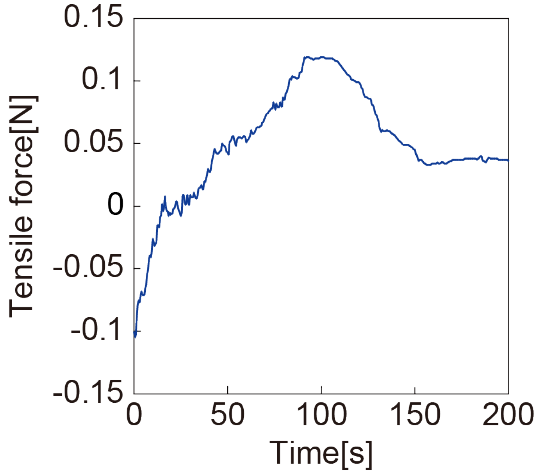

Supplementary Figure S13. Relationship between time and measured tensile force when peeling off the ion gel-LM bilayer wiring with a width of 2 mm from the glass substrate.

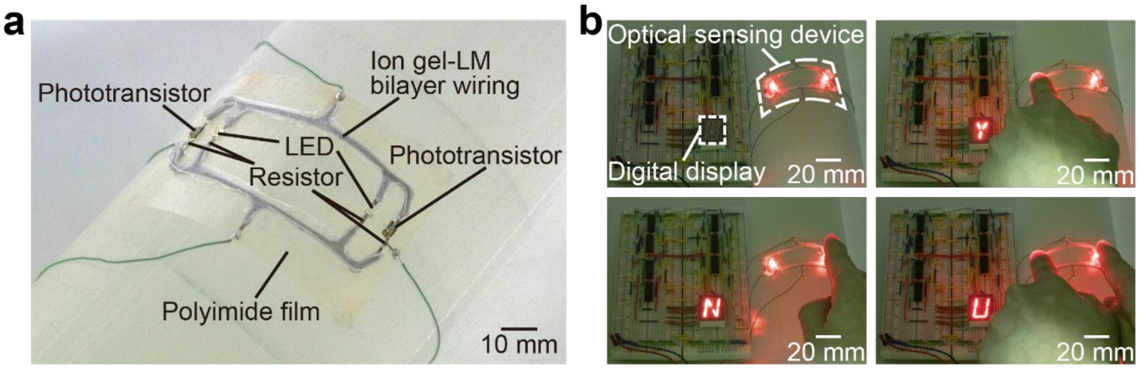

Supplementary Figure S14. Operation of the optical sensing circuit on the flexible substrate when bent. (a) Magnified view of the constructed circuit. (b) Four-character patterns controlled by hand movements using the external device connected to the circuit.

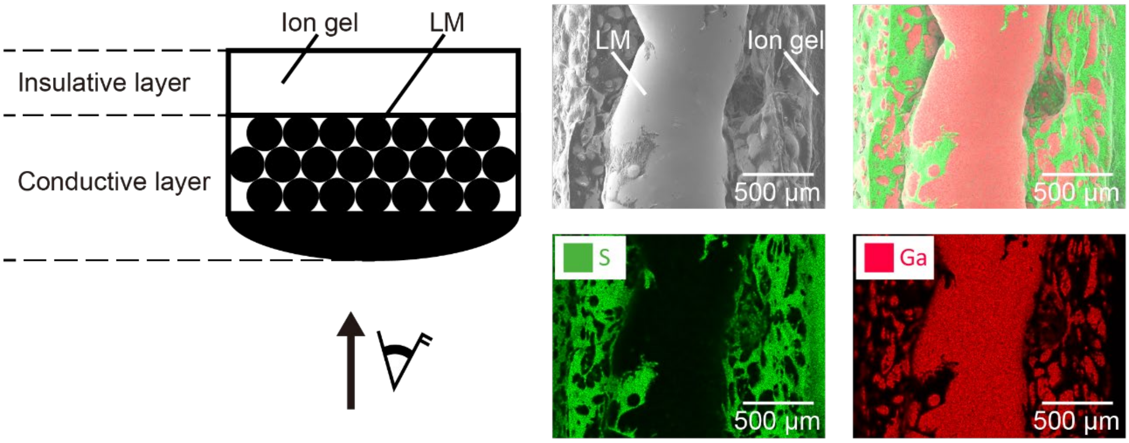

Supplementary Figure S15. Bottom images of the ion gel-LM bilayer wiring using scanning electron microscopy-energy dispersive X-ray spectroscopy (SEM EDX).

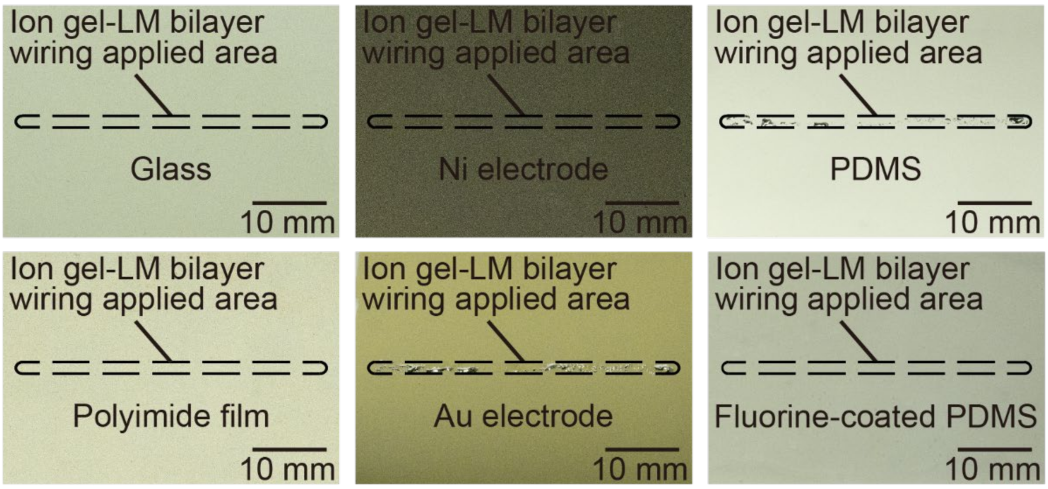

Supplementary Figure S16. Adhesion of LM when the ion gel-LM bilayer wiring with LM particle size of 167 μm and LMGC layer thickness of 0.260 mm is applied to glass, polyimide film, Ni electrode, Au electrode, PDMS, and fluorine-coated PDMS. LM adheres only to the Au electrode and PDMS.

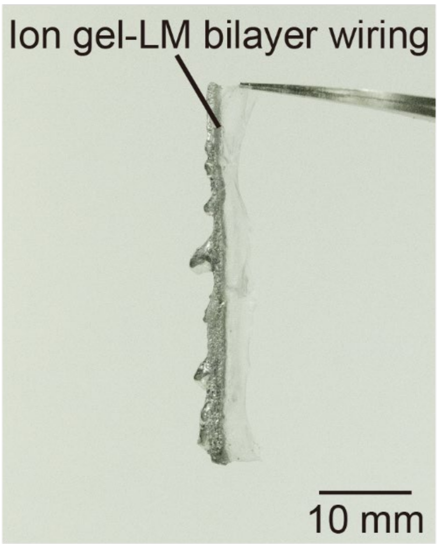

Supplementary Figure S17. LM retained at the bottom of the ion gel-LM bilayer wiring with LMGC layer thickness of 1.04 mm.

Supplementary Table S1. Comparison between the LM wiring technology in this study and conventional LM wiring technologies.

	Encapsulation	Design flexibility	Conductivity	Resolution
Direct writing[24, 25, 26]	×	○	3.4×10^{6} S/m	1.9 μm
Screen printing[27, 28]	×	○	3.46×10^{6} S/m	200 μm
Laser sintering[30, 31]	×	○	3×10^{6} S/m	200 μm
Injection[15, 34]	○	×	3.46×10^{6} S/m	67 μm
LM-silicone composite[35, 36]	○	○	1.4×10^{2} S/m	400 μm
This study	○	○	1.58×10^{6} S/m	404 μm

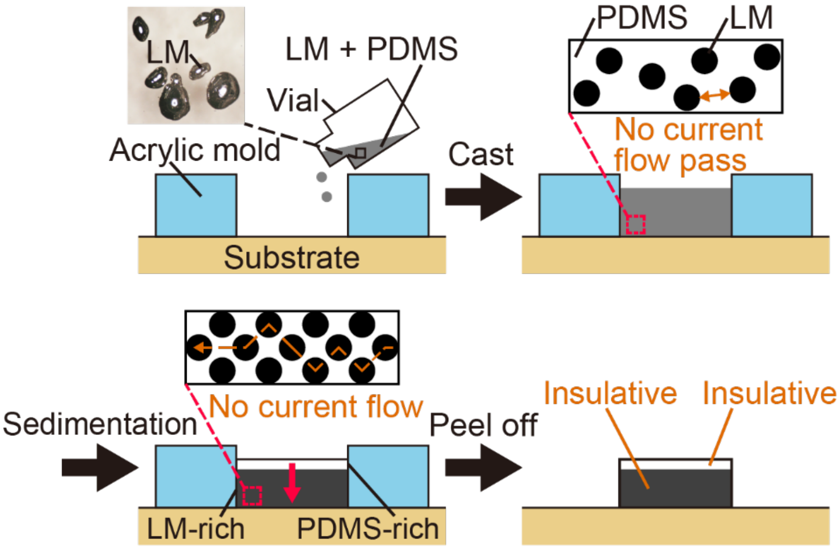

Supplementary Figure S18. Fabrication method of PDMS-LM composite.

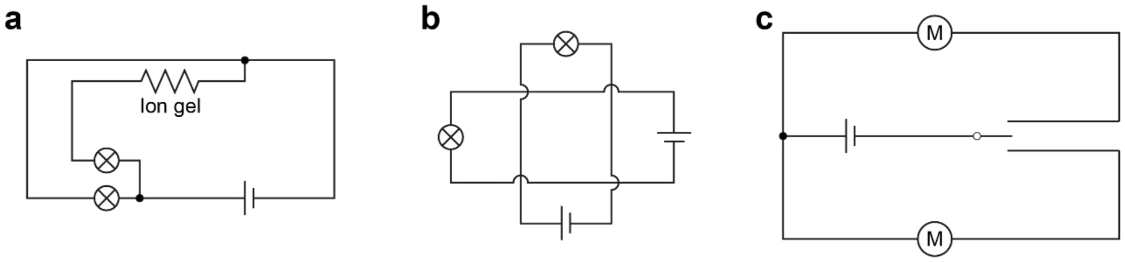

Supplementary Figure S19. Circuit diagrams of the demonstrations performed using ion gel-LM bilayer wiring. (a) Circuit diagram for verifying the insulative layer performance of ion gel-LM bilayer wiring. (b) Circuit diagram of 3D wiring using ion gel-LM bilayer wiring. (c) Circuit diagram for switching using ion gel-LM bilayer wiring.

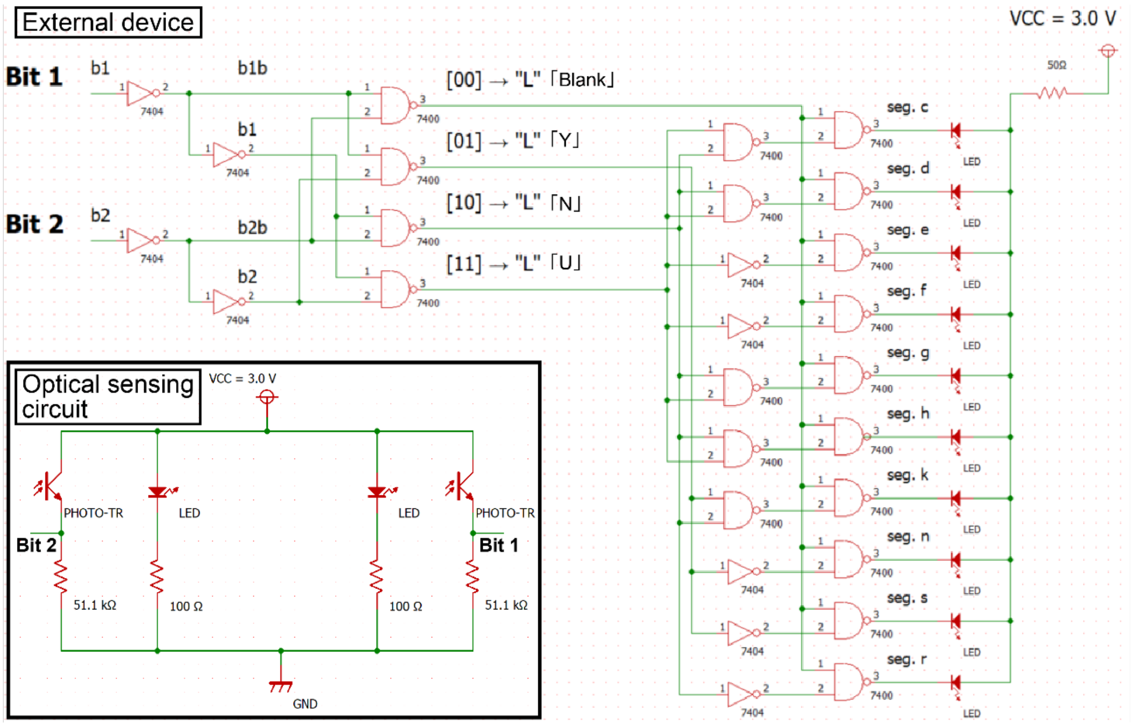

Supplementary Figure S20. Circuit diagrams of the optical sensing circuit and external device.

Supplementary Video S1. Independent lighting of LEDs in 3D wiring using ion gel-LM bilayer wiring.

Supplementary Video S2. Circuit switching using ion gel-LM bilayer wiring.

Supplementary Video S3. Operation of the optical sensing circuit and external device displays four-character patterns.
